# Avascular Necrosis in Patients With Cushing Syndrome

**DOI:** 10.1210/jcemcr/luaf001

**Published:** 2025-02-05

**Authors:** Noa Tal, Serguei Bannykh, Thomas Learch, Adam N Mamelak, Odelia Cooper

**Affiliations:** Division of Endocrinology, Diabetes, and Metabolism, Department of Medicine, Cedars-Sinai Medical Center, Los Angeles, CA 90048, USA; Department of Pathology, Cedars-Sinai Medical Center, Los Angeles, CA 90048, USA; Department of Radiology, Cedars-Sinai Medical Center, Los Angeles, CA 90048, USA; Pituitary Center, Cedars-Sinai Medical Center, Los Angeles, CA 90048, USA; Pituitary Center, Cedars-Sinai Medical Center, Los Angeles, CA 90048, USA

**Keywords:** Cushing disease, avascular necrosis, hypercortisolism

## Abstract

Cushing syndrome (CS) results from prolonged exposure to excess glucocorticoids, leading to a range of clinical manifestations including avascular necrosis (AVN), a rare complication of CS. Although AVN is often associated with exogenous glucocorticoid treatment, it can occur in endogenous CS but may be unrecognized because of its rarity and possibly from a subclinical presentation. We describe a case of a 71-year-old male with florid Cushing disease who initially presented with bilateral hip AVN and later developed bilateral shoulder AVN despite achieving biochemical remission following transsphenoidal surgery and adjuvant stereotactic photon radiosurgery. AVN in endogenous CS is underreported, and guidance on routine screening is lacking. Our case underscores the importance of considering AVN in patients with CS, especially in those with persistent or recurrent joint symptoms and markedly elevated cortisol levels. Early detection of AVN is crucial as it can lead to irreversible joint damage and disability if untreated. Screening strategies should be explored to identify high-risk patients who are diagnosed with CS for timely intervention, thereby preventing long-term morbidity associated with AVN.

## Introduction

Cushing syndrome (CS) results from prolonged exposure to excess glucocorticoids, either from exogenous glucocorticoids or endogenous sources. In endogenous CS, hypercortisolism may be due to an ACTH-dependent process, most often from a corticotroph adenoma in Cushing disease (CD) or from ectopic ACTH secretion from neuroendocrine tumors or other solid tumors such as small cell lung carcinoma. On the other hand, ACTH-independent CS is mainly driven from adrenal pathology including adrenal adenomas, adrenocortical carcinomas, adrenal hyperplasia, and primary pigmented micronodular disease [1]. The presenting symptoms and signs of CS include hypertension, diabetes mellitus, weight gain, facial plethora, dorsocervical fat pads, muscle weakness, and osteoporosis, most of which may be detected on physical examination or diagnosed biochemically. A less common symptom is avascular necrosis (AVN) of bone tissue [1, 2], which can present with pain or point tenderness of the hip or other joints as well as present subclinically [3].

AVN of the hip results from compromised blood supply to the bone tissue and usually impacts the hips and shoulders. This leads to necrosis of hematopoietic cells, adipocytes, and osteocytes. Subsequently, bone repair processes are activated, with differentiation of mesenchymal cells into osteoblasts to build new bone and hematopoietic stem cells into osteoclasts to remove necrotic tissue. However, because of impaired bone resorption and formation, subchondral fractures eventually occur [4]. Exogenous glucocorticoid treatment is 1 of the most common causes of AVN and may account for up to 38% of atraumatic AVN and is dose dependent [5]. Glucocorticoid treatment is theorized to cause AVN through increased systemic lipids, leading to compromised perfusion to the femoral head resulting from fat emboli or external lipocyte compression, as well as alterations in the inflammatory cytokines resulting in osteoclast activation and osteoblast apoptosis [4, 6]. Compared to exogenous glucocorticoid treatment, AVN caused by endogenous hypercortisolism is not frequently reported nor is it screened for on diagnosis of CS.

We describe a patient who presented with bilateral hip AVN in the context of florid CD. We aim to highlight this presenting feature to heighten awareness for screening for this progressive condition, which can potentially lead to joint damage, loss of mobility, and long-term disability.

## Case Presentation

A 71-year-old male with medical history of active tobacco use and obstructive sleep apnea was diagnosed with new-onset hypertension during an annual health visit. He was started on antihypertensive medications (losartan, hydrochlorothiazide, and spironolactone) by his primary care doctor, but the hypertension remained uncontrolled. Over the course of 2 months, the patient developed progressive lower extremity edema and was started on furosemide, which led to hypokalemia and was subsequently discontinued. He clinically deteriorated, with progressive anasarca and dyspnea, and then developed acute left eye ptosis and diplopia and was admitted to the hospital. The patient also endorsed irritability, mood swings, easy bruising, low libido, increased appetite, 30-lb weight gain, and bilateral hip pain.

## Diagnostic Assessment

**Figure 1.**
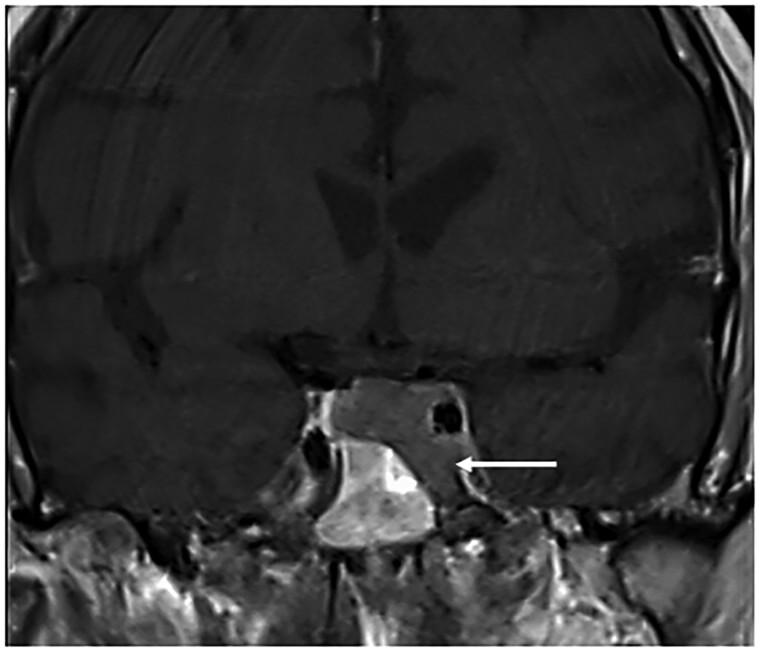
Pituitary magnetic resonance imaging (MRI) with gadolinium, using T1-weighted, turbo spin-echo revealed sequence revealed a 16 × 20 × 16 mm macroadenoma invading the left cavernous sinus (white arrow).

**Figure 2.**
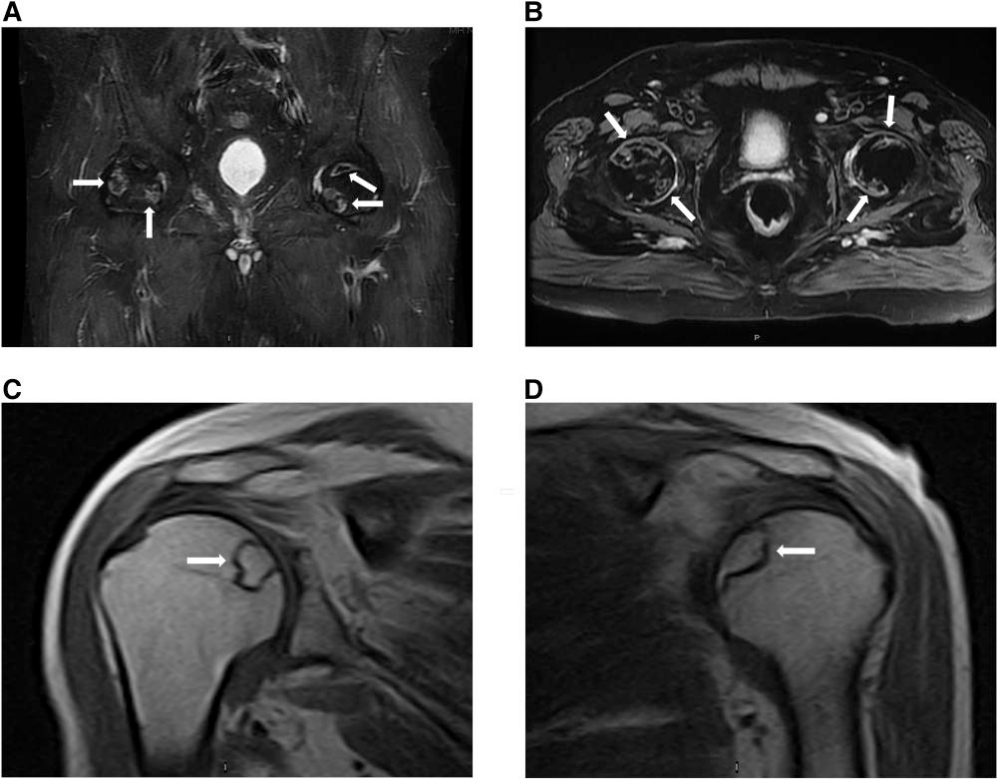
Coronal inversion recovery image bilateral hips demonstrates geographic lesions bilateral femoral heads with serpentine borders consistent with bilateral femoral head bone infarcts. No subchondral collapse or arthritic changes identified (A). Axial proton density with fat saturation image bilateral hips demonstrates geographic lesions bilateral femoral heads with serpentine borders consistent with bilateral femoral head bone infarcts. No subchondral collapse or arthritic changes identified (B). Coronal T1 image of the right shoulder demonstrates geographic lesion medial humeral head with serpentine border consistent with bone infarct. No subchondral collapse or arthritic changes identified (C). Coronal T1 image of the left shoulder demonstrates geographic lesion medial humeral head with serpentine border consistent with bone infarct. No subchondral collapse or arthritic changes identified (D) (white arrows).

Physical examination was significant for oral candidiasis, dorsocervical fat pad, facial plethora, proximal muscle weakness, and bilateral hip tenderness. Testing confirmed ACTH-dependent CS with elevated 24-hour urine free cortisol of 1116 μg/24 hours (30788.21 nmol/24 hours) and 1171.9 μg/24 hours (32330.38 nmol/24 hours) (normal reference range, 3.5-45 μg/24 hours; 96.56-1241.46 nmol/24 hours) and ACTH of 173 pg/mL (38.06 pmol/L) and 112 pg/mL (24.64 pmol/L) (normal reference range, 7.2-63 pg/mL; 1.58-13.86 pmol/L) on 2 separate occasions. He had hypogonadotropic hypogonadism with total testosterone levels of 41 ng/dL (1.42 nmol/L) (normal reference range, 250-1100 ng/dL; 8.68-38.17 nmol/mL) and suppressed LH and FSH at <0.2 mIU/mL (<0.2 IU/L) (normal reference range, 0.6-12.1; 0.6-12/1.1 IU/L) and 0.2 mIU/mL (<0.2 IU/L) (normal reference range, 1.0-12.0 2 mIU/mL; 1.0-12.0 2 IU/L) respectively, whereas the remaining pituitary hormones were normal, although IGF-1 was low normal at 66 ng/mL (8.65 nmol/L) (normal reference range, 7.2-63 pg/mL; 1.58-13.86 pmol/L). He also had new-onset diabetes mellitus with glycated hemoglobin of 8% (<5.7%) (Table 1). Imaging of the lungs showed a 15-mm solid noncalcified nodule in the posterior right upper lobe concerning for neoplasm. Pituitary magnetic resonance imaging (MRI) revealed a 16 × 20 × 16 mm macroadenoma invading the left cavernous sinus (Fig. 1). Additionally, pelvis computed tomography (CT) scan demonstrated bilateral avascular necrosis of the capital femoral epiphysis without evidence of fracture or subchondral collapse (Fig. 2A and 2B).

**Table 1.** Laboratory evaluation of the patient at presentation

Lab	Value	Reference Range Conventional units (Système International units)
ACTH	173 pg/mL (38.06 pmol/L)	7.2-63 pg/mL (1.58-13.86 pmol/L)
24-h urine free cortisol	1116 μg/24 h (30,788.21 nmol/24 h)	4.0-55.0 μg/24 h (110.35-1517.34 nmol/24 h)
Total testosterone	41 ng/mL (1.42 nmol/L)	250-1100 ng/mL (8.68-38.17 nmol/L)
Free testosterone	12.3 pg/mL (0.07 nmol/L)	30.0-135.0 pg/mL (0.17-0.79 nmol/L)
LH	<0.2 mIU/mL (<0.2 IU/L)	0.6-12.1 mIU/mL (0.6-12.1 IU/L)
FSH	0.2 mIU/mL (0.2 IU/L)	1-12 mIU/mL (1-12 IU/L)
Prolactin	9.6 ng/mL (9.6 μg/L)	3.5-19.4 ng/mL (3.5-19.4 μg/L)
TSH	0.746 mIU/L	0.450-5.330 mIU/L
Free T4	0.66 ng/dL (8.49 pmol/L)	0.61-1.60 ng/dL (7.85-20.59 pmol/L
IGF-1 *Z* score	66 ng/mL (8.65 nmol/L) −0.9	34-245 ng/mL (4.45-32.09 nmol/L) −2.0 to +2.0
HbA1c	8.2%	<5.7%

Abbreviations: Hb A1c, hemoglobin A1C.

## Treatment

**Figure 3.**
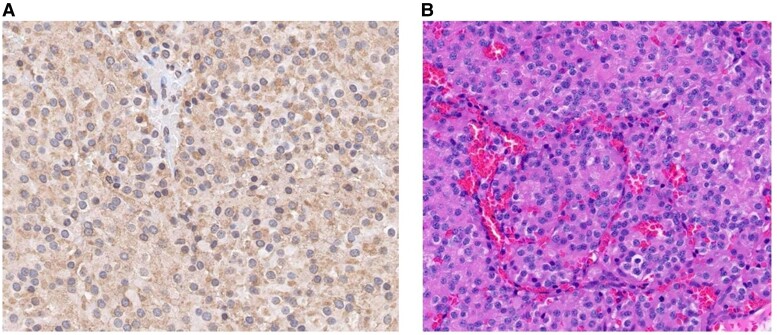
Hematoxylin and eosin (A) and adrenocorticotropic hormone (B) stained sections show oval nuclei with “salt and pepper” chromatin and granular, ACTH-positive cytoplasm. Original magnification 250×.

Prophylactic treatment was started with subcutaneous heparin for anticoagulation and trimethoprim-sulfamethoxazole for opportunistic infections. Orthopedic evaluation did not recommend acute intervention for the hip AVN. Given the pituitary macroadenoma on imaging and left cranial nerve VI palsy, it was determined that the patient likely had CD, so he underwent transsphenoidal surgery. Surgical pathology confirmed the adenoma was ACTH positive, sparsely granulated, with Ki-67 index of 4%, and without increased mitotic activity (Fig. 3).

## Outcome and Follow-up

Due to ongoing hypercortisolism (Table 2) and residual tumor in the left cavernous sinus, the patient underwent adjuvant treatment with stereotactic photon radiosurgery at a dose of 13 Gy targeted to the left cavernous sinus and was started on osilodrostat, an oral, reversible inhibitor of 11β-hydroxylase that drives the final step of cortisol synthesis and aldosterone synthase, which converts 11-deoxycorticosterone to aldosterone [7]. The starting dose of osilodrostat was 2 mg twice per day. As the patient developed nausea, lack of appetite, and malaise with decreasing cortisol levels, osilodrostat was reduced to 1 mg daily, and he was started on hydrocortisone replacement therapy on week 11 postoperatively (Table 3). Ultimately, both osilodrostat and hydrocortisone were discontinued following normalization of cortisol levels. Regarding the rest of the hormonal deficiencies, his total testosterone and IGF-1 levels improved to levels of 483 ng/dL (16.76 nmol/L) and 99 (12.97 nmol/L), respectively, and he did not require hormone replacement therapy. Clinically, the patient improved with resolution of his hypertension and diabetes and achieved a 38-lb weight loss. Additionally, his diplopia improved and his hip pain resolved without any restriction in mobility. However, 1 year postoperatively, the patient developed bilateral shoulder pain. MRI of the shoulders demonstrated subchondral changes in the right humeral head (Fig. 2C) and a linear area of subchondral change involving the left humeral head (Fig. 2D) consistent with AVN, as well as a bilateral high-grade supraspinatus tear and acromioclavicular joint osteoarthritis. He was treated with an intraarticular methylprednisolone 40-mg injection to both shoulders, with subsequent improvement of the pain and joint mobility. He also underwent a coronary artery bypass graft surgery for 3-vessel disease. The patient has otherwise maintained normal urine and salivary cortisol levels off osilodrostat or hydrocortisone, and 1 year after surgery, the ACTH (cosyntropin) stimulation test was normal. The pulmonary nodule has remained stable on serial imaging.

**Table 2.** Postoperative cortisol and ACTH levels

		Postoperative day					
**Lab**	**Reference Range** Conventional units (Système International units)	1	2	2	3	4	5
Morning cortisol	3.7-19.4 μg/dL (102.08- 535.21 nmol/L)	26 μg/dL (717.29 nmol/L)	21.5 μg/dL (593.14 nmol/L)	6 μg/dL (165.53 nmol/L)	8.1 μg/dL (223.46 nmol/L)	16.4 μg/dL (452.44 nmol/L)	21.5 μg/dL (593.14 nmol/L)
ACTH	7.2-63.3 pg/mL (1.58- 13.93 pmol/L)		72 pg/mL (15.84 pmol/L)			62 pg/mL (13.64 pmol/L)	

**Table 3.** Titration of osilodrostat treatment based on cortisol levels

		Postoperative week							
**Lab**	**Reference range** Conventional units (Système International units)	8	9	11	13	15	18	22	24
ACTH	7.2-63.3 pg/mL (1.58-13.93 pmol/L)	95.6 pg/mL (21.03 pmol/L)	131 pg/mL (28.82 pmol/L)	58.8 pg/mL (12.94 pmol/L)	79.3 pg/mL (17.45 pmol/L)	79.9 pg/mL (17.58 pmol/L)	73.4 pg/mL (16.15 pmol/L)	62 pg/mL (13.64 pmol/L)	71.5 pg/mL (15.73 pmol/L)
Morning cortisol	3.7-19.4 μg/dL (102.08-535.21 nmol/L)	23.9 μg/dL (659.35 nmol/L)	18.8 μg/dL (518.65 nmol/L)	6.6 μg/dL (182.08 nmol/L)	4.5 μg/dL (124.15 nmol/L)	3.3 μg/dL (91.04 nmol/L)	2.4 μg/dL (66.21) nmol/L	8.2 μg/dL (226.22. nmol/L)	4.1 μg/dL (113.11 nmol/L)
LNSC	<0.010-0.090 μg/dL (0.28-2.48 nmol/L)		0.615 μg/dL (16.97 nmol/L)		0.058 μg/dL (1.60 nmol/L)	0.041 μg/dL (1.13 nmol/L)			0.041 μg/dL (1.13 nmol/L)
UFC, 24-h	5-64 μg/24 h (137.94-1765.63 nmol/24 h)	246 μg/24 h (6786.65 nmol/24 h)	226 μg/24 h (6234.89 nmol/24 h)		2 μg/24 h (55.18. nmol/24 h)				
Osilodrostat dose		2 mg BID	2 mg BID	2 mg AM 3 mg PM	2 mg BID	2 mg AM 1 mg PM	1 mg BID	1 mg daily	Oslidrostat discontinued

Abbreviations: BID, twice per day; LNSC, late night salivary cortisol; UFC, urine free cortisol.

## Discussion

Our patient exhibited pronounced hypercortisolism secondary to CD, with bilateral hip AVN as 1 of the presenting symptoms. Despite achieving biochemical remission of the disease and resolution of other associated symptoms, the patient was later diagnosed with bilateral shoulder AVN.

AVN caused by endogenous hypercortisolism is seldom documented, and routine screening for it is not typically conducted during the diagnosis of CS. However, AVN has been reported to be a presenting symptom in several case reports or may manifest years after the initial diagnosis [8]. Reported causes of AVN in endogenous CS include pituitary adenomas, adrenal adenomas or carcinomas, adrenal hyperplasia, or neuroendocrine tumors [8‐23] (Table 4), with some cases of AVN associated with severe hypercortisolism [10, 15]. Other risk factors associated with AVN include hip trauma, femoral fractures, hip dislocation, systemic lupus erythematosus in the setting of concomitant corticosteroid treatments, or vasculitis, sickle cell disease, hypercoagulability, Gaucher disease, hyperlipidemia or hypertriglyceridemia, hyperuricemia, hematological malignancies, antiretroviral medications, alcohol use, and exogenous steroid treatment [4]. Our patient had no history of hip trauma or other aforementioned comorbidities. Furthermore, during presentation, his lipid levels were normal, with low-density lipoprotein cholesterol of 89 mg/dL (<130 mg/dL) and triglycerides of 97 mg/dL (<150 mg/dL). Therefore, it is likely that his bilateral hip and shoulder AVN was caused by severe endogenous hypercortisolism.

**Table 4.** Published cases of avascular necrosis in patients with endogenous hypercortisolism

First author, year	Age (y)/sex	Time of diagnosis in relation to CS diagnosis	AVN related symptoms	Imaging modality	Imaging description	Diagnosis	Treatment
Salazar D, 2021 [15]	38 F	3 y prior to diagnosis	Right hip pain	MRI	Right hip joint effusion and synovitis Flattening of the femoral head-Subcortical edema	Adrenal adenoma	Right hip arthroplasty
Madell SH, 1964 [16]	41 F	1 month before diagnosis	Right shoulder pain	X-ray	Increased density of the right humeral head with spotty areas of radiolucency Early flattening and beginning of fragmentation	Adrenal adenoma	Osteotomy
Anand A, 2022 [21]	47 M		Bilateral hip pain	MRI	Necrosis of bilateral femur heads	adrenocortical carcinoma	
Belmahi N, 2018 [9]	28 F		Progressive limping and right hip pain	MRI	Right femoral head AVN	Pituitary adenoma	Right total hip replacement
Wicks I, 1987 [10]	39 M	18 months before diagnosis	Progressive hip pain and stiffens	X-ray Bone scan	Lucent and sclerotic regions within flattened femoral heads Some loss of articular cartilage	Pituitary adenoma	Conservative management
Koch C, 1999 [11]	30 F		Sudden onset of severe left hip pain	MRI	Abnormal high intensity signal changes in the bone marrow of the left femoral head Joint effusion Stage 2 AVN	Pituitary adenoma	Immediate core decompression surgery with decongestion of the left femoral head
Premkumar M, 2013 [12]	26 F	2 y after pituitary surgery for Cushing, while on replacement steroid therapy	Progressive bilateral hip pain resulting in difficulty in walking	MRI	Bilateral multiple bony infarcts in the proximal femur and distal femur Femoral head collapse fractures -Stage 2 avascular necrosis	Pituitary adenoma	
Bauddh N, 2022 [13]	24 M	2 y prior to diagnosis	Progressive left hip pain and difficulty in walking	X-ray MRI	Left femoral head AVN	Pituitary adenoma	Planned for surgery of hip AVN
Joseph A, 2022 [14]	21 F	1 y prior to diagnosis	Bilateral hip joint pain	X-ray MRI	Ill-defined mixed sclerotic and lytic pattern of the femoral heads Cortical disruption of the round contour Low signal intensity in the subchondral region of the femoral necks on T1-weighted images	Pituitary adenoma	Planned for total hip replacement. Bisphosphonates.
Pazderska A, 2016 [19]	36 F		Right leg pain	MRI	Bilateral AVN of the femoral heads Left femoral head with early bone fragmentation	Bilateral primary pigmented micronodular adrenal disease	Spontaneous healing of AVN after adrenalectomy.
Papadakis G, 2017 [22]	55 F			MRI PET/CT 68Ga-DOTATATE	Bilateral AVN Bone marrow edema extending to the intertrochanteric area Mild subchondral femoral head collapse of the left hip Increased activity in bilateral femoral heads and in the bone marrow consistent with edema Mild left femoral head collapse	Ectopic ACTH- secreting tumor	
Phillips K, 1986 [8]	24 F	4.5 y after diagnosis	Right femoral AVN	X-ray	Flattening and sclerosis of femoral head	Cushing disease	
	25 F	4 y after diagnosis	Right femoral AVN		Subchondral lucency		
	43 F	8 mo after diagnosis	Right humeral AVN		Sclerosis and flattening of articular surface of humeral head		
	61 F	11 y after diagnosis	Left femoral AVN and bilateral humeral heads		Cortical indistinctness and subchondral lucency Left humeral head flattening and sclerosis		
Cerletty J, 1973 [20]	54 M	3 mo before diagnosis	Right femoral head fracture	X-ray	Bilateral subchondral sclerosis of the femoral heads Some narrowing of the joint space on the left Infraction of the margin of the right femoral head Femoral neck fracture.	Bilateral adrenal cortical hyperplasia	Total hip joint arthroplasty
Ha J-S, 2019 [18]	36 F	2 y before diagnosis	2 mo left hip restricted range of motion	X-ray MRI	Right femoral head with areas of hyperlucency and surrounding sclerosis Subtle changes in the shape of the articular surface Bilateral femoral head osteonecrosis -Increased amount of joint fluid and bone marrow edema in the left hip Right femoral head necrosis	Adrenal cortical adenoma	Total hip replacement
Takada, J, 2004 [17]	55 F		Intense right hip pain and a limp	MRI	Low-intensity band on T1-weighted images Stage 2 AVN.	Adrenal adenoma	Total hip arthroplasty
Modlinger RS, 1972 [23]	69 F		Increased pain of right shoulder	X-ray	Bilateral shoulders with aseptic necrosis of the humeral heads	Ectopic ACTH secretion NET form pancreatic tumor	

Abbreviations: AVN, avascular necrosis; F, female; M, male; MRI, magnetic resonance imaging; NET, neuroendocrine tumor.

AVN can result in irreversible femoral head collapse, leading to severe limitation in movement, reduced joint functionality, and decreased quality of life [24]. Initially, patients may be asymptomatic or endorse nonspecific pain when presenting with AVN and may not be diagnosed until an advanced stage when they develop more severe pain and disability [25]. In a meta-analysis assessing the prevalence of AVN in patients with systemic lupus erythematosus, including those who received corticosteroid treatment, asymptomatic AVN was detected in 29% of patients and symptomatic disease was noted in 9% [26]. AVN can diagnosed with MRI or CT imaging. Although noncontrast MRI has higher sensitivity and specificity in detecting early stages of the disease, CT is comparable to MRI in more advanced stages. Ancillary imaging modalities include plain radiography, positron emission tomography, and bone scan [27].

Staging of AVN relies on radiologic features and size of lesions. In earlier stages, imaging can be normal (stage 0) or with subtle abnormalities on MRI or bone scan and normal radiography (stage 1). As the disease progresses, structural changes, including cystic and sclerotic changes (stage 2), subchondral collapse (stage 3), flattening of the femoral head (stage 4), joint narrowing and acetabular changes (stage 5), and, finally, advanced degenerative changes (stage 6) can be detected on most imaging modalities.

Management of early stages of AVN includes observation or conservative weight-bearing management, medical therapy with bisphosphonates, anticoagulation therapy, statins, and vasodilators. Invasive procedures such as mesenchymal stem cells implantation, osteotomy, surgical joint decompression, and total hip replacement are reserved for more advanced stages [28]. Indeed, AVN accounts for approximately 10% of total hip replacements in the United States [29]. Staging has prognostic implications for treatment options and disease outcomes. Early-stage disease, when diagnosed and treated, can often regress, and be cured. Conservative measures, medical treatment, biophysical stimulation, extracorporeal shockwave therapy, or core decompression, can prevent femoral head collapse and further hip arthroplasty. On the other hand, late-stage disease, characterized by joint collapse, is irreversible and often requires joint replacement [30].

Although actual prevalence rates of AVN in endogenous CS is unknown, one should consider screening for AVN in this high-risk population, particularly in patients showing markedly elevated cortisol levels, as in our case. Such an approach would facilitate the early identification of individuals who would benefit from earlier medical or surgical interventions, thereby preventing permanent joint destruction and chronic disability.

## Learning Points

- AVN can be a complication of endogenous hypercortisolism.
- AVN may present asymptomatically or with nonspecific symptoms such as joint pain.
- AVN can affect multiple joints, including hips and shoulders, and its early diagnosis relies on MRI or CT imaging.
- Early detection and intervention for AVN are crucial to prevent irreversible joint damage and disability.
- Screening for AVN in patients with CS should be considered to enable timely intervention and prevent long-term complications, particularly in patients with hip or shoulder pain and severe hypercortisolism.

## Contributors

All authors made individual contributions to authorship. N.T. and O.C. were involved in the diagnosis and management of the patient and manuscript submission. S.B. was involved in the histopathology section and preparation of histology images. T.L. was involved in the interpretation and preparation of the radiology images. A.N.M. was responsible for the patient's surgery and treatment plan. All authors reviewed and approved the final draft.

## Data Availability

Data sharing is not applicable to this article as no datasets were generated or analyzed during the current study.

## Abbreviations

AVN: avascular necrosis
CD: Cushing disease
CS: Cushing syndrome
CT: computed tomography
MRI: magnetic resonance imaging
